# The art of combining neuroanatomy and microsurgical skills in modern neurosurgery

**DOI:** 10.3389/fneur.2022.1076778

**Published:** 2023-01-12

**Authors:** Juan Carlos Ahumada-Vizcaino, Raphael Wuo-Silva, Manuel Moreno Hernández, Feres Chaddad-Neto

**Affiliations:** ^1^Department of Neurology and Neurosurgery, Universidade Federal de São Paulo, São Paulo, SP, Brazil; ^2^Department of Neurosurgery, Beneficência Portuguesa Hospital, São Paulo, SP, Brazil

**Keywords:** laboratory, microsurgical skills, neuroanatomy, white matter fibers, human placenta, cadaveric models, vascular neurosurgery

## Abstract

Neurosurgical training outside the operating room has become a priority for all neurosurgeons around the world. The exponential increase in the number of publications on training in neurosurgery reflects changes in the environment that future neurosurgeons are expected to work in. In modern practice, patients and medicolegal experts demand objective measures of competence and proficiency in the growing list of techniques available to treat complex neurosurgical conditions. It is important to ensure the myriad of training models available lead to tangible improvements in the operating room. While neuroanatomy textbooks and atlases are continually revised to teach the aspiring surgeon anatomy with a three-dimensional perspective, developing technical skills are integral to the pursuit of excellence in neurosurgery. Parapharsing William Osler, one of the fathers of neurosurgical training, without anatomical knowledge we are lost, but without the experience and skills from practice our journey is yet to begin. It is important to constantly aspire beyond competence to mastery, as we aim to deliver good outcomes for patients in an era of declining case volumes. In this article, we discuss, based on the literature, the most commonly used training models and how they are integrated into the treatment of some surgical brain conditions.

## 1. Introduction

The introduction of the operating microscope to neurosurgery led to the age of precise handling of sub-millimeter anatomical structures to treat pathology while limiting or averting injury to nearby critical structures. This age of microsurgery has witnessed the mapping of CSF cisterns and advances in the operating corridors such as the opening of the choroidal fissure and new techniques to perform bypasses in previously hard-to-reach parts of the skull (1).

**Figure 1:**
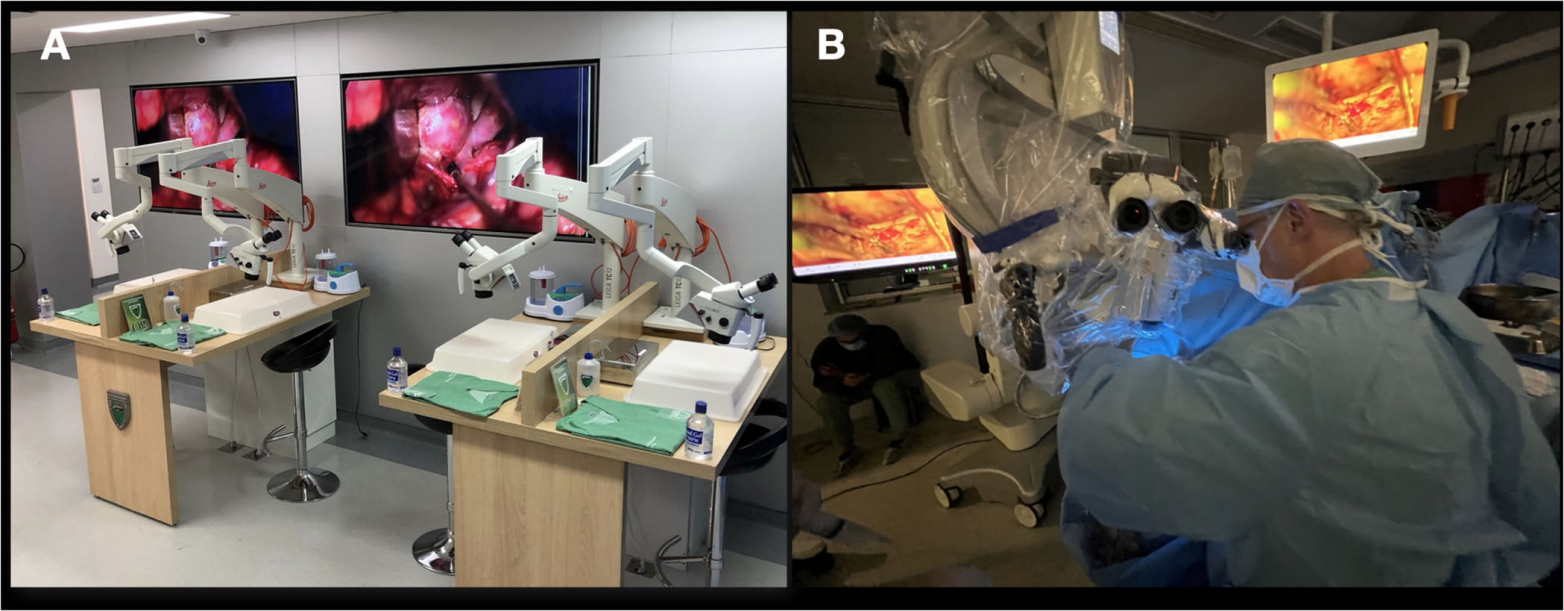
**(A)** The laboratory; **(B)** must imitate the scenario that happens in the operating room.

For each of these advances, we have found specific aspects of microvascular laboratory training that hone the required skill. Appropriate training is required under controlled conditions, outside the operating room (Figure 1) (1).

We will describe the most common training models used around the world, the purposes of each of the training models, and how can all be used and incorporated into targeted microsurgery training.

## 2. Applied skills in specific training models from case illustration

### 2.1. Posture and hand position

**Figure 2:**
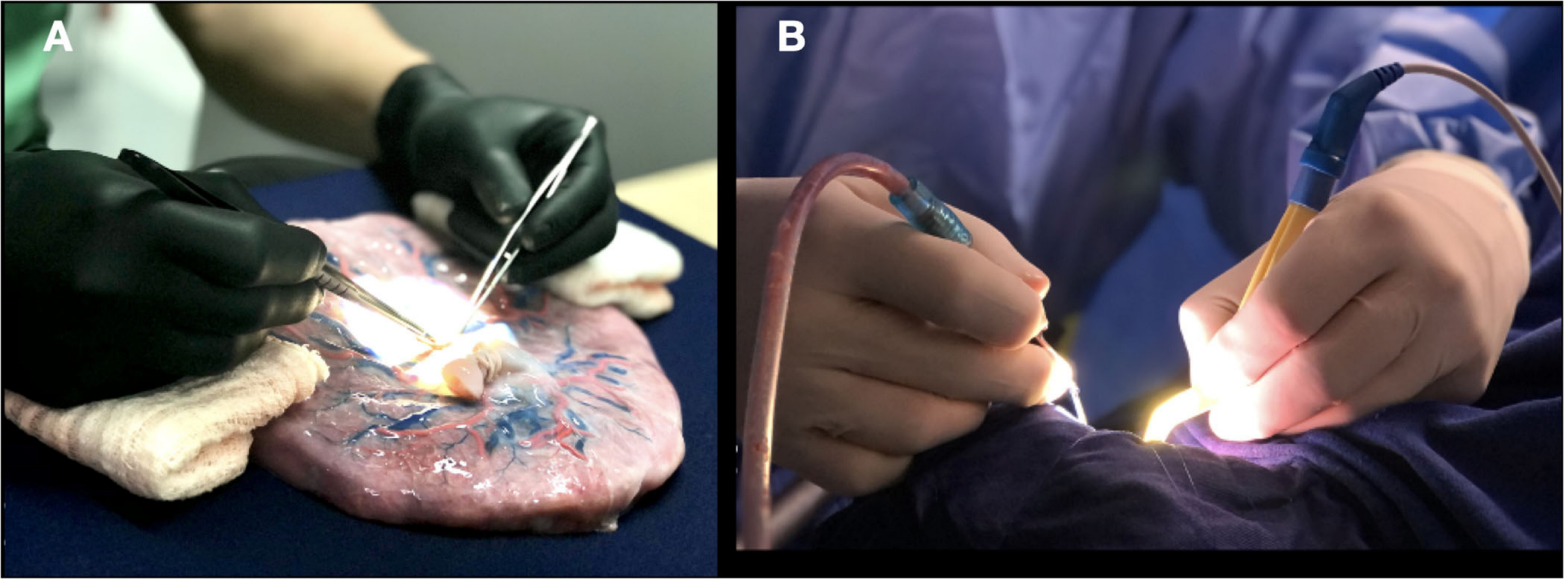
**(A)** Positioning the hands in the laboratory; **(B)** Surgical field.

**Figure 3:**
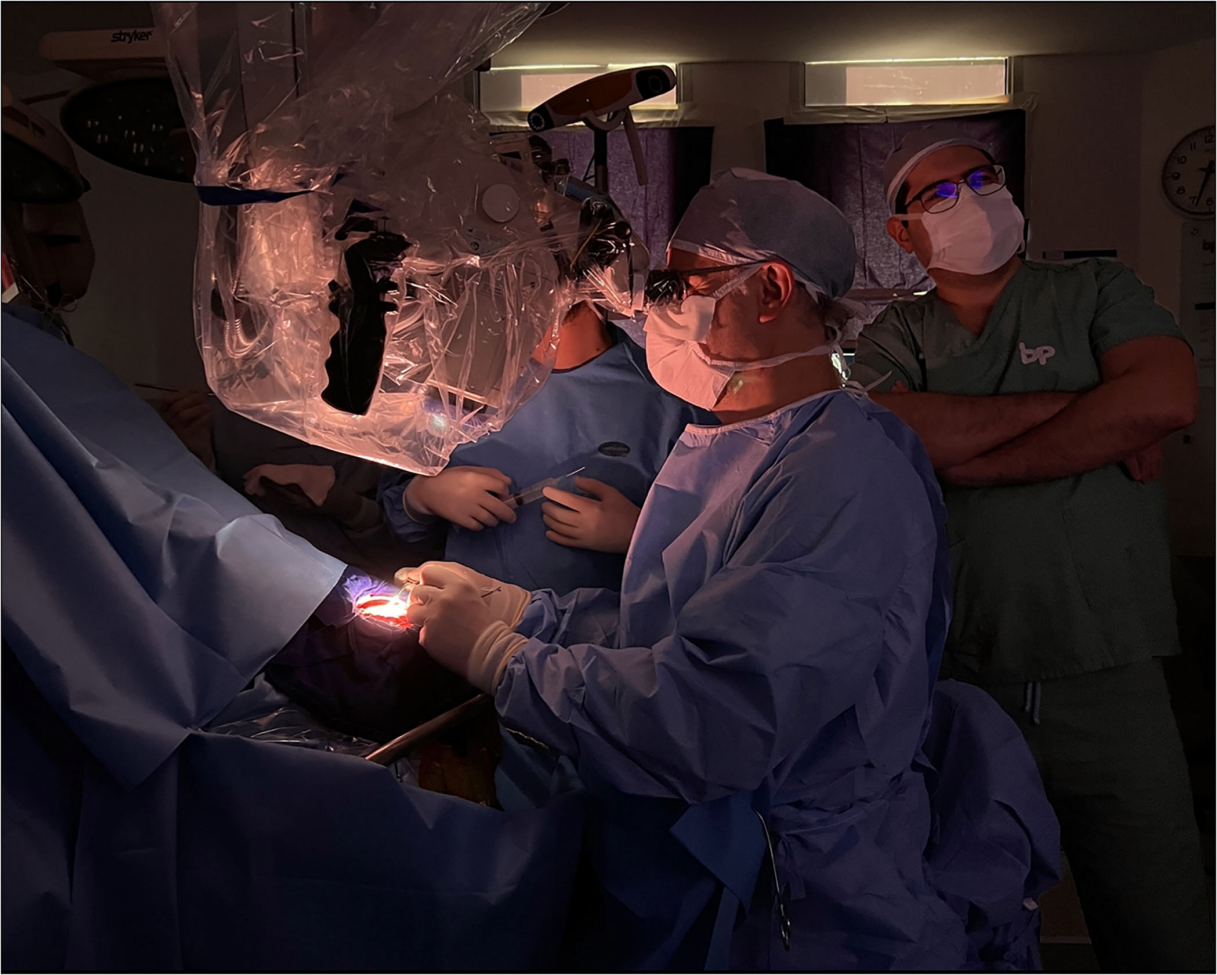
Ergonomic posture goes from the spine, arms, and support of the hands around the craniotomy.

The available training opportunities start with ensuring the posture, the position of our hands, and the use of instruments that are optimized and rehearsed for the task. An ergonomic posture includes aligning the spine and resting the arms and hands to reduce the effort required to use instruments (Figures 2, 3). A bad posture at any level will cause a sequence of corrections that will exacerbate tremors and ultimately affect the performance of the microsurgical technique.

### 2.2. The cerebral sulci, gyri, and ventricles

When the neurosurgeon makes a craniotomy and opens the dura mater, it makes eye contact with the surface of the brain. This can be intimidating at the beginning. There are no names or arrows indicating which structure is seen. For this reason, it is important to study previously with cadaveric specimens. Therefore, prior knowledge of structures such as sulcus, gyrus, and ventricles structures such as sulcus, gyrus, and ventricle elements and their relations with supratentorial and deep anatomic structures help the neurosurgeon to plan the best corridors to treat neurosurgical conditions (2, 3).

**Figure 4:**
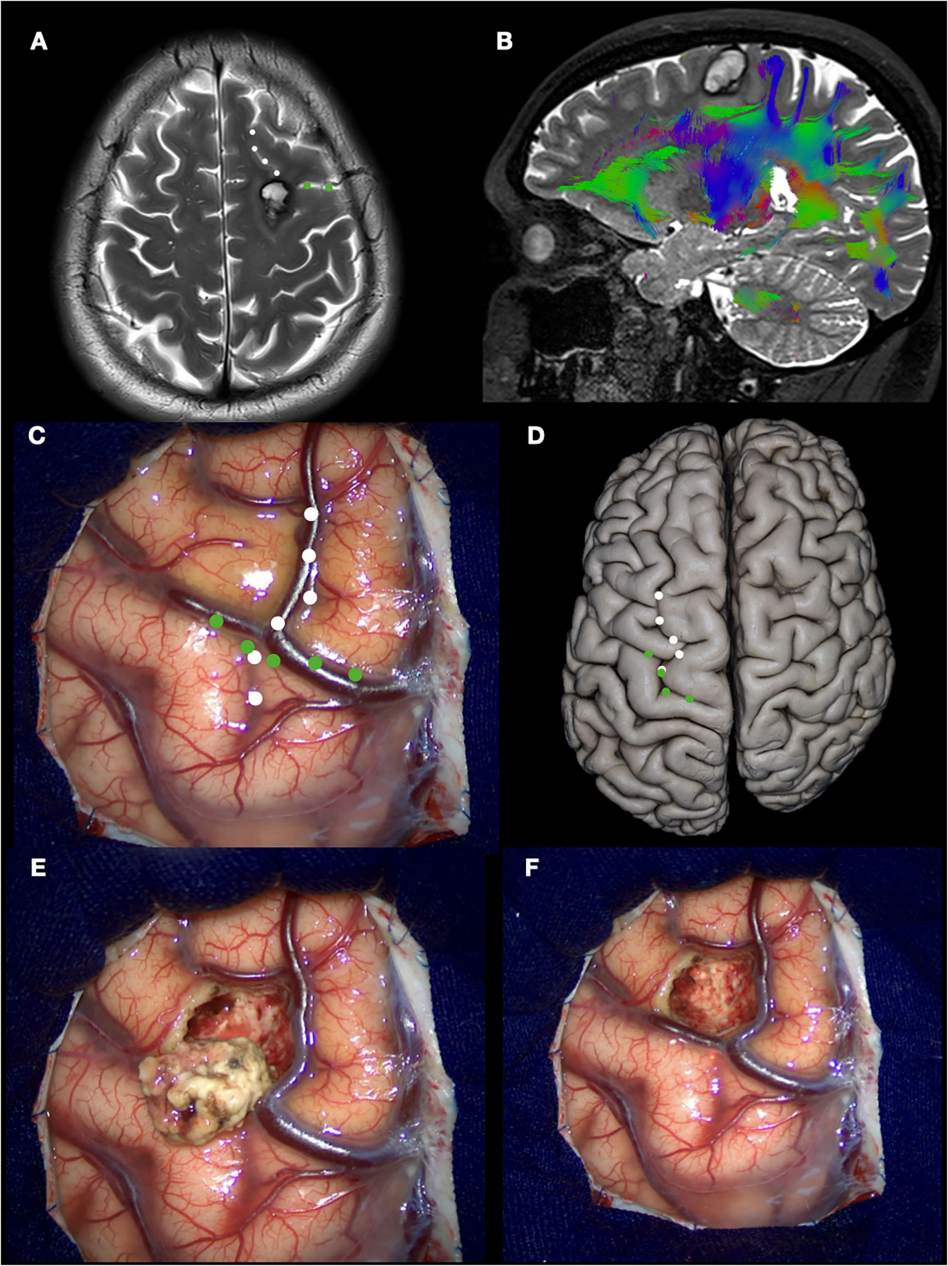
Left prefrontal cavernous malformation. **(A)** T2 MRI showing a hemorrhage between superior, middle, and precentral gyri (white dotted line: superior frontal sulcus; green dotted line: precentral sulcus); **(B)** DTI tractography demonstrating the relation with the underlying tracts; **(C)** intraoperative view. The hemosiderin staining over the middle frontal gyrus is seen, indicating the previous hemorrhage; **(D)** Anatomic specimen demonstrating the same landmarks in **(C)**; **(E)** Cavernous malformation resected; **(F)** Final inspection.

For example, in this case, a 38-year-old man presented a focal onset motor seizure in the right hand. During the MRI investigation, a cavernous malformation with a recent hemorrhage located in the superior frontal sulcus, closely related to the precentral gyrus, was diagnosed. Surgery was indicated, achieving complete resection. As we know, in Penfield's homunculus, the motor representation of the hand is located at the intersection of the superior frontal sulcus with the precentral sulcus. Figure 4 shows the anatomical and surgical images. The advantage in this situation is that in the human specimen, we can study the disposition of the gyri, sulci, and the distance between the structures, and this will help and facilitate the neurosurgeon for the localization of this specific area, sparing the limits of the resection.

To improve the study of the specimens, in 2008, Mattos et al. described a step-by-step dissection (standardized routine sequence of cuts) that allows the identification of all the important structures without the risk of damaging other parts of the brain in the attempt to show one in particular. This is a priority because new specimens are rarely available in most laboratories around the world. Therefore, guiding the dissection and preparation of these specimens are very important with the purpose of showing the largest number of structures as possible, with minimal damage to the piece (3).

### 2.3. White matter fiber tract dissection

Once the neurosurgeon learns the disposition of the surface anatomy of the brain, he must advance into the unshaped magma that characterizes the white matter of the brain in dealing with many lesions, including primary or secondary brain tumors, intraventricular lesions, cavernomas, arteriovenous malformations, hippocampal sclerosis, and others. For this reason, knowledge of the organization that underlies the white matter, although complex and not completely elucidated, is of significant neurosurgical importance.

In basic structure, the white matter is composed of myelinated fibers grouped into three types of tracts or fasciculi: association fibers interconnecting different cortical regions of the same hemisphere, commissural fibers interconnecting the two hemispheres across the median plane, and projection fibers passing up and down the neuraxis, and connecting the cortex with caudal parts of the brain and the spinal cord (4).

During the 1930s, white matter tracts began to assume relevance for neurosurgery, especially after Cajal's work. In 1934, at the University of Basel under Eugen Ludwig, Klingler developed a new method of dissection based on a freezing technique for the brain tissue that eloquently revealed the white matter tracts (5).

Klingler wrote and advised those wishing to learn neuroanatomy:

“In order to prevent disappointments, it must be emphasized that the production of a true and instructive brain preparation is not an occupation for beginners. An indispensable requirement is a good knowledge of the gross anatomy of the brain to the extent indicated by the atlas of Edward Flatau (1895), and by the work of Gustaf Retzius (1896, 1898). The second requirement is practiced hands, trained on dissections easier to accomplish; the third is patience and perseverance. The instruments are of secondary importance” (5).

Due to this method, microneurosurgery evolved rapidly, creating interest in developing new techniques in the management of intrinsic injuries. Later, with the introduction of diffusion tensor imaging (DTI)-based tractography, it was possible to correlate and detail the brain architecture with anatomical studies of white fiber dissection (6).

The use of all these tools led the neurosurgeon to treat pathologies, such as gliomas, in an anatomical and functional manner, sometimes achieving radical resections without generating significant neurological damage, thereby improving the quality and life expectancy of the patient (7, 8).

Therefore, white fiber dissection allows young neurosurgeons the ability to project three-dimensional images of deep brain structures onto the brain surface, which, together with functional neuroimaging studies and intraoperative monitoring (brain mapping), help them to develop, including creating new surgical approaches (9–12).

**Figure 5:**
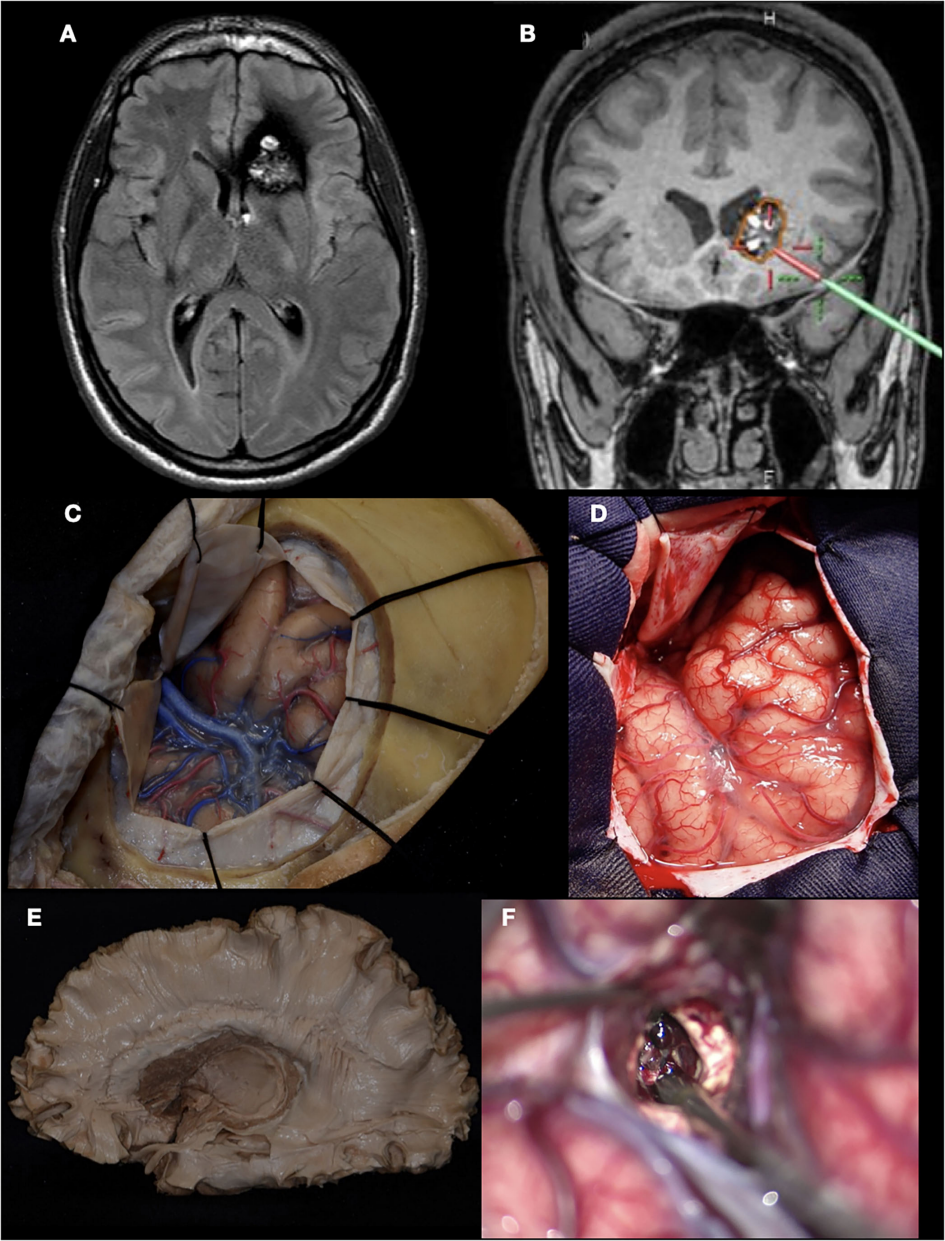
Cavernous malformation of the left caudate head. **(A)** MRI (FLAIR) showing the classic “popcorn” appearance of a cavernoma located in the left caudate nucleus; **(B)** Neuronavigation demonstrating the planned trajectory; **(C, D)** Pterional craniotomy performed in the lab (cadaveric specimen) and in the operating room; **(E)** White matter fiber dissection showing the localization of the caudate head; **(F)** Microscope view demonstrating the characteristic “berry” appearance of the cavernous malformation.

Currently, with all this knowledge established, the next case is about a 21-year-old man presented with a sudden intense headache without an altered state of consciousness. An MRI was performed, revealing a cavernoma with recent bleeding in the head of the caudate nucleus. A pterional craniotomy and resection of the cavernoma were performed (13). Figure 5 shows the key points that include surgical planning, use of auxiliary tools (neuronavigation), craniotomy design, and superficial and deep anatomy, as well as the characteristic intraoperative appearance of the cavernoma. Knowing the underlying fascicules that surround the head of the caudate nucleus allows us to plan a shorter and safer approach to this region. Specifically, in this case, the route chosen was through the ascending ramus of the Sylvian fissure, which in the deepest part corresponds with the anterior limiting sulcus of the insula. With this approach, we can respect the horizontal part of the superior longitudinal fasciculus and uncinate fasciculus, as we can see in the anatomical piece of Figure 5E, with a little amount of transection of the inferior fronto-occipital fasciculus to reach the head of the caudate nucleus. The three-dimensional orientation and direction of the resection begins at the laboratory and is confirmed with neuronavigation.

### 2.4. Vascular training

At this point in our review, the neurosurgeon has only had to work with the brain itself. But once we add the vascular structures, the degree of complexity begins to increase. During surgery, it becomes vital to know how to handle blood vessels, both arteries and veins, and work between them, avoiding injuring them. Furthermore, it requires a long time of training to reach a level of expertise in surgical technique (14).

In recent years, endovascular therapy has gained considerable ground in the treatment of a large part of vascular diseases. In fact, in some centers and countries, it has displaced microneurosurgery as the first treatment option. This is largely due to the following two situations: the lower learning curve and the decrease in the training of new neurosurgeons with specific training in cerebrovascular surgery (15).

Therefore, microsurgical training should begin with the management of vascular structures in controlled settings. We will expose the gradual sequence of training to develop the appropriate microsurgical skills.

### 2.5. Human placenta

The human placenta is a maternal–fetal structure made up of a larger fetal portion and a smaller maternal portion. The fetal surface has a fetal chorion (amniotic membrane) and a rich vascular component with vessels of 1–6 mm in diameter. The anterior cerebral artery has a diameter of 1–3 mm and the middle cerebral artery has a diameter between 2.4 and 4.6 mm. On the contrary, the vertebral artery has a diameter of 0.92–4.09 mm, and the posterior cerebral and posterior inferior cerebellar arteries have diameters between 0.65 and 1.78 mm. These characteristics make the placenta an excellent tool for the development of the microsurgical technique (16).

The fetal chorion (chorionic membrane) looks similar to the arachnoid. Therefore, one of the first steps in training is to separate and dissect this membrane.

**Figure 6:**
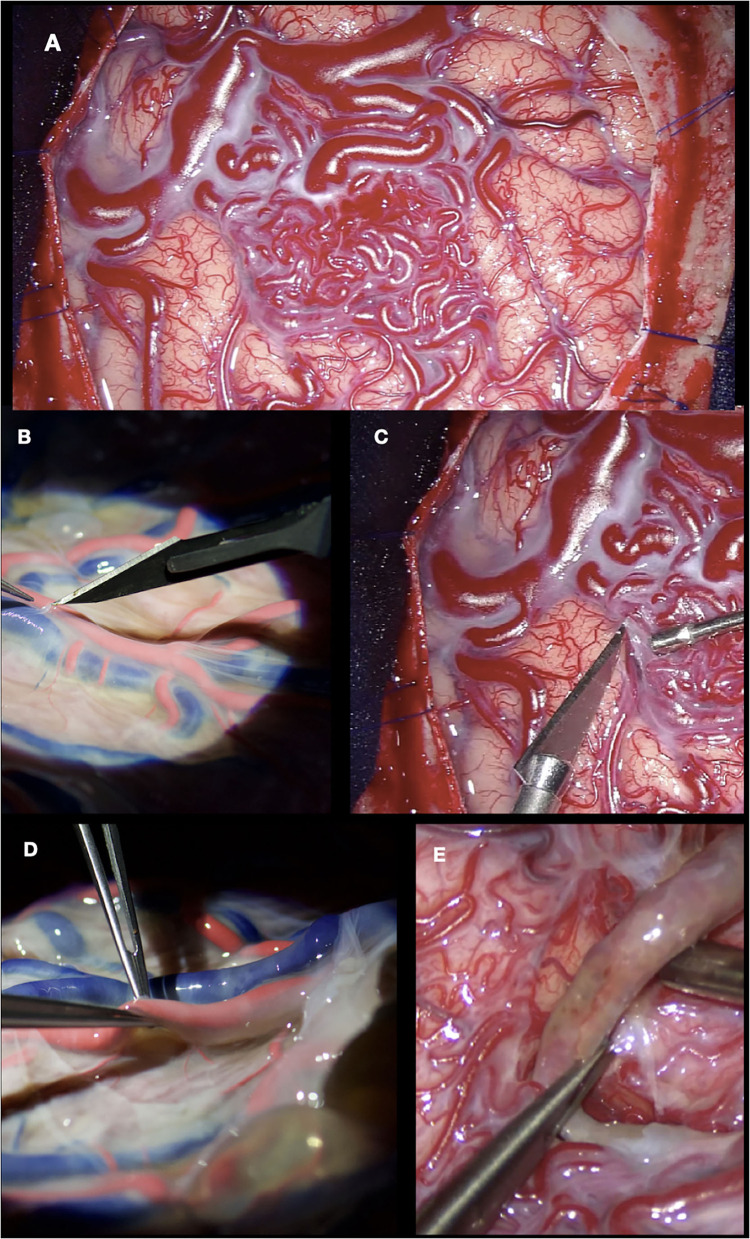
**(A, B)** Dissection of the superficial temporal artery in cadaveric specimen and surgery, respectively; **(C)** Right temporoparietal AVM exposed; **(D)** Separating the chorionic membrane from the placental vessels; **(E)** Dissection of the AVM from the parenchyma, maintaining the arachnoid plane.

For example, in this case, a 33-year-old woman presented with a history of intermittent chronic headaches, without any associated neurological deficit. She was diagnosed with a right temporoparietal arteriovenous malformation and underwent microsurgical treatment. In Figure 6, we can see how the use of techniques learned and practiced in the placenta can be extrapolated in the operative field. The dissection of the chorionic membrane and subsequently the separation of the vessels from the chorionic stroma allows us to perform the circumferential dissection of the AVM, maintaining the arachnoid plane, and also the skeletonization of both the feeding arteries and the drainage veins.

Microsurgical training is mandatory for neurosurgeons performing brain bypass, usually performed on single-vessel simulators, such as live animals, chicken wings, or synthetic models, with limited vessel caliber variety. Furthermore, brain bypass can be performed in cadaveric specimens (17, 18).

As mentioned earlier, the placenta has several vessels of different calibers, and due to their distribution, it is possible to perform end-to-end, end-to-side, and side-to-side anastomoses. This type of anastomosis can be performed with or without the infusion of dye solution into the vessels; however, it is preferable to perform with solution, since this allows better handling of the vessel (19).

Separating the arteries from the chorionic stroma must be done carefully, since the main vessels contain small branches that, if cut, generate dye leakage and consequently loss of pressure within the vessel. In a real patient surgery, these small branches are coagulated. In the case of the placenta, mini clips can be placed. The aneurysm can be made and intraoperative rupture can be simulated (19).

**Figure 7:**
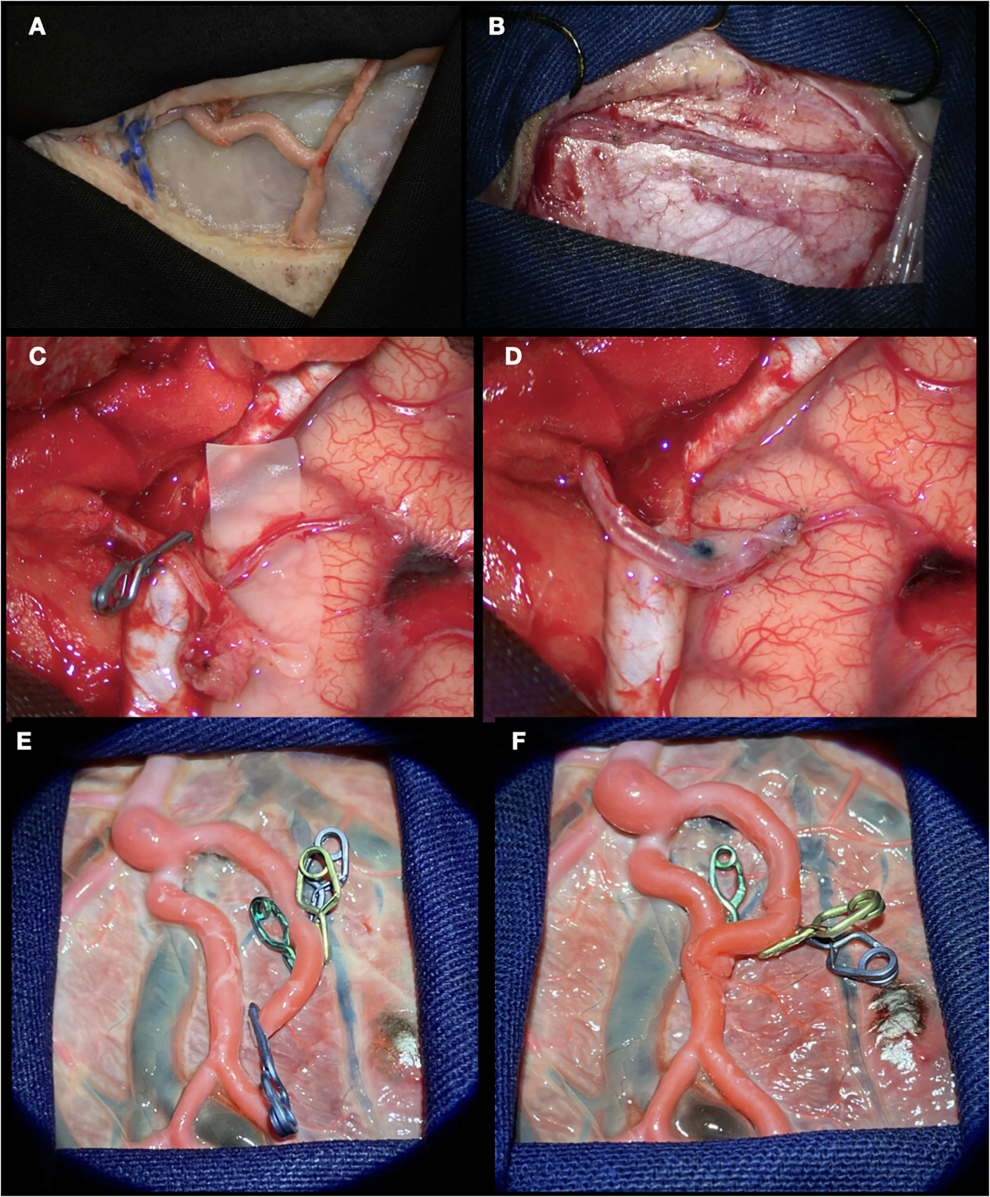
STA-MCA bypass. **(A, B)** Dissection of the superficial temporal artery performed in cadaveric specimen and patient, respectively; **(C)** STA and MCA skeletonized; **(D)** End-to-side anastomosis performed; **(E)** Branch of placental artery dissected and sectioned. The use of clips in the small branches prevents dye leakage; **(F)** End-to-side anastomosis done.

In this illustrative case, a 32-year-old woman with repeated episodes of left hemiparesis and speech disturbances, with partial recovery after rehabilitation was diagnosed with Moyamoya disease and underwent direct revascularization (STA-MCA bypass). Figure 7 shows how an end-to-side anastomosis made in the placenta has the same characteristics as one made in a real patient. The use of clips during placenta training prevents the loss of the dye while we perform the suture. A low-flow bypass was chosen. Vascular bypasses are performed relatively infrequently even at experienced centers (10–20/year). The speed and fluency with which the procedure is executed are enhanced by practice in the laboratory, including in the hands of master surgeons.

### 2.6. Cadaveric models

Cadaveric models undoubtedly offer the best visuospatial scenario in which all microsurgical techniques can be performed. In 2002, Emad et al. described a cadaveric model with dynamic flow both in the blood vessels and in the subarachnoid space, which is currently known as the “Perfusion-based Human Cadaver Simulation Training Model.” This type of model offers the advantage of experiencing the sensation of being in a situation with a real patient. During the opening of the skin, the neurosurgeon in training faces “bleeding” not only from the skin flap but also at all levels of the approach (20).

Once the approach has been carried out, it is possible to create artificial aneurysms in different locations, opening the arteries and suturing vessel grafts (mainly veins). This allows us to reproduce and practice placing a clip, a procedure that can be repeated as many times as necessary. The different techniques of anastomosis (bypass) can also be practiced in these models (21–23).

## 3. Integrating neuroanatomical knowledge and microsurgical skills

All the theoretical and technical aspects mentioned earlier have a common purpose to offer surgical treatment for a given pathology with a lower risk of damage to uninvolved structures and a potential cure. Anatomical knowledge complements surgical technique and vice versa. If any of these is not adequate, the possibility of failure is high. Therefore, the improvement and systematized structuring at the time of performing surgery are essential for an optimal outcome.

We present another two cases where neuroanatomy and the techniques learned in the laboratory are applied in the treatment of cerebrovascular disease and brain tumors.

**Figure 8:**
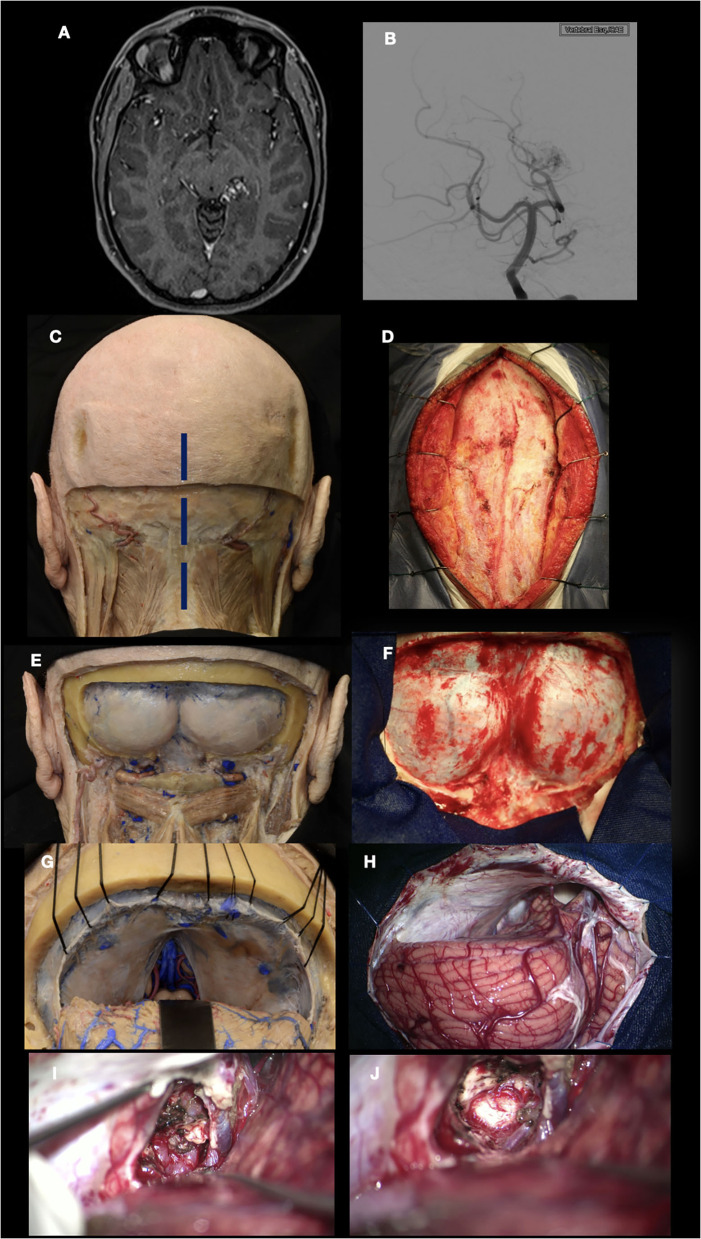
Left parahippocampal gyrus arteriovenous malformation. **(A, B)** MRI and DSA show an AVM located in the left parahippocampal gyrus, supplied by branches of the P3 segment from the posterior cerebral artery. **(C–F)** Suboccipital approach in the cadaveric specimen and surgical field; **(G, H)** Supracerebellar infratentorial approach performed; **(I)** After the resection of the tentorium, the AVM was exposed within the parahippocampal gyrus; **(J)** AVM resected. The thalamus pulvinar is seen in the deepest part.

In the first case, a 29-year-old woman presented with 3 months of headaches and seizures. MRI and DSA revealed an AVM located in the left parahippocampal gyrus, posterior to the pulvinar thalamus. The patient underwent microsurgical treatment in a semi-sitting position, and the approach chosen was a supracerebellar infratentorial with transtentorial resection. Figure 8 details the correlation between a cadaveric specimen and the steps during the surgery (24, 25). As we can see, training the exposure in the laboratory before in a real patient let the neurosurgeon learn how to position the hands, open the skin, the extension the craniotomy, the space generated during the supracerebellar infratentorial approach, which structures the neurosurgeon will find after opening the tentorium, and which instruments he needs to improve the treatment of the condition, in this case, an AVM. Perfectioning this approach in a cadaveric specimen has an advantage in terms of handling the working distance that no other training model could offer.

**Figure 9:**
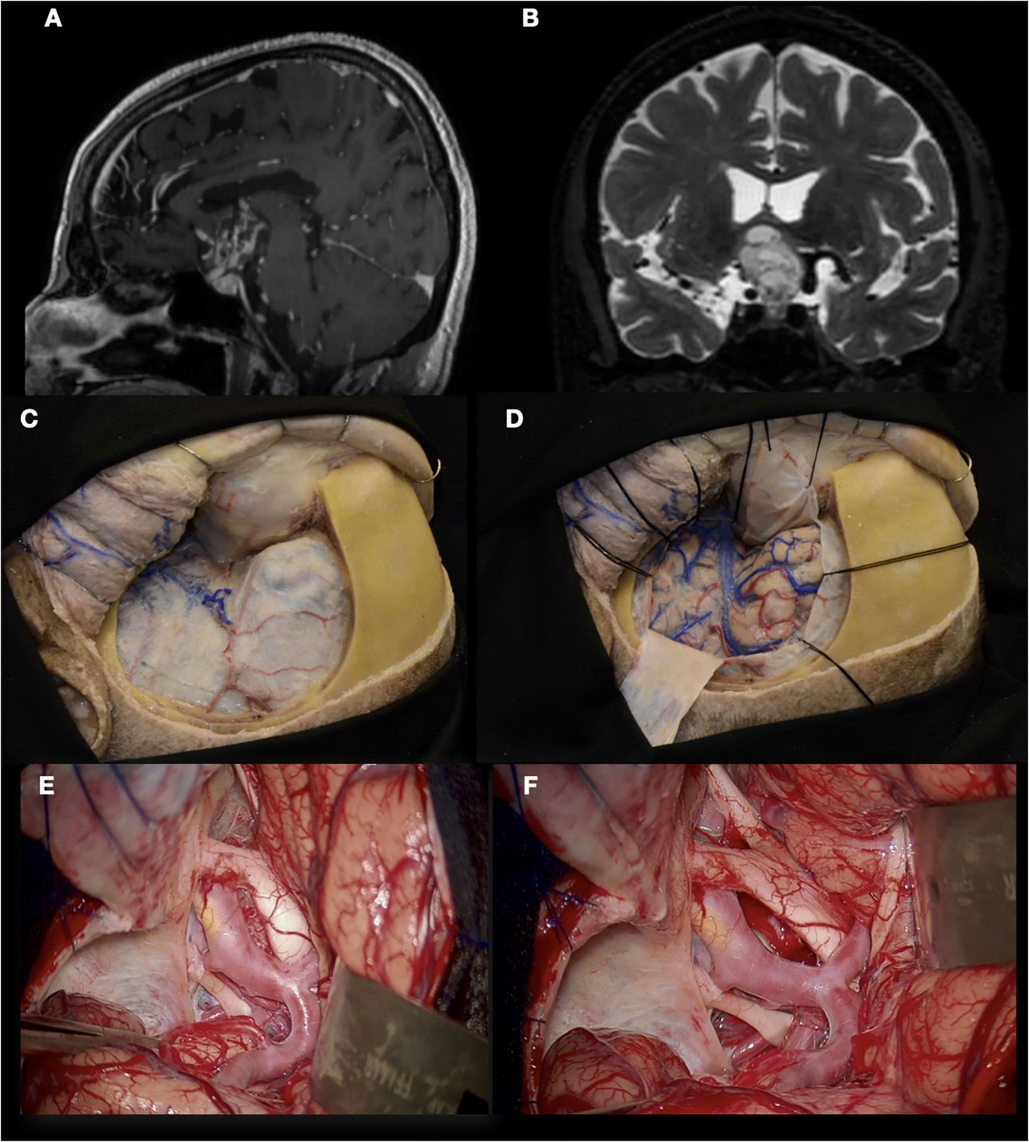
Craniopharyngioma. **(A, B)** T1 and T2 images show a craniopharyngioma that extends into the third ventricle; **(C, D)** Orbitozygomatic (OZ) craniotomy performed in a cadaveric specimen; **(E)** Surgical view that offers the OZ craniotomy. Craniopharyngioma can be seen in the opticocarotid triangle. Optic nerve and Chiasm are being displaced by the tumor superiorly; **(F)** Overview after craniopharyngioma was resected.

In the last case, a 70-year-old man presented with a history of chronic headaches and decreased visual acuity. A craniopharyngioma extending to the third ventricle was diagnosed. Orbitozygomatic craniotomy and microsurgical resection were performed. It is important to know that when suprasellar lesions are in contact with the floor of the third ventricle or beyond, orbitozygomatic craniotomy is indicated to improve vision from inferior to superior (Figure 9) (26). The arachnoid dissection of the Sylvian cistern, which allows the neurosurgeon to separate the frontal lobe from the temporal lobe, exposing the subfrontal, transsylvian, and pretemporal corridors, allowing to work in the interoptic, opticocarotid, and carotid-oculomotor spaces with a microsurgical technique for tumor resection, are some of the advantages offered by previous training in the laboratory.

## 4. Discussion

An exponential number of neurosurgical training models are introduced each year in the neurosurgical literature. These can broadly be divided into models that focus on the acquisition of anatomical or technical knowledge, the practice of technical skills, and those that allow skills to be acquired in an anatomical and clinically relevant context.

The case illustrations highlighted specific aspects of complex microsurgical procedures that can be rehearsed in the laboratory to improve performance. While the discussion is styled as a perspective rather than a scientific study, most readers will accept that good practice and training are related to proficiency and mastery. We hope to provoke readers to evaluate new models of surgical training by investigating how closely they enable the user to practice aspects of surgery away from the intended environment while maintaining the constraints an individual is likely to encounter.

The present discussions have been limited to open microsurgery but remain relevant for the adoption of new technological advances, such as the exoscope and extended use of the endoscope in neurosurgery.

## 5. Conclusion

Sound anatomical knowledge complements surgical technique and vice versa with the deficiency in one or the other likely to impact the outcome of the surgery. Constant rehearsal of microsurgical skills while maintaining the anatomical context and operating room challenges are recipes for developing true mastery in the art of modern neurosurgery.

Maintaining a focus on the operating room during the development of new adjuncts to training will ensure our efforts on the laboratory bench transfer seamlessly to the operating room and produce consistent outcomes for our patients.

## Author contributions

JA-V: preparation, editing, and illustration of the manuscript. MH: writing and editing. RW-S: writing, reviewing, and editing the manuscript. All authors contributed to the article and approved the submitted version.
